# Diabetes alters the protein secretome of human adipose‐derived stem cells and promotes tumorigenesis in hepatic cancer cells

**DOI:** 10.1002/ctm2.823

**Published:** 2022-06-02

**Authors:** Miriam Ejarque, Joan Sabadell‐Basallote, Ester Benaiges, Catalina Núñez‐Roa, Eduardo Sabido, Eva Borras, Erik Llacer, Antonio Zorzano, Joan Vendrell, Sonia Fernández‐Veledo

**Affiliations:** ^1^ Unitat de Recerca Hospital Universitari de Tarragona Joan XXIII Institut d'Investigació Sanitària Pere Virgili Tarragona Spain; ^2^ CIBER de Diabetes y Enfermedades Metabólicas Asociadas (CIBERDEM) Instituto de Salud Carlos III Madrid Spain; ^3^ Departament de Medicina i Cirurgia, Facultat de Medicina Universitat Rovira i Virgili Tarragona Spain; ^4^ Proteomics Unit Centre de Regulació Genòmica Barcelona Institute of Science and Technology Barcelona Spain; ^5^ Departament de Ciències Experimentals i de la Salut Universitat Pompeu Fabra Barcelona Spain; ^6^ Institute for Research in Biomedicine (IRB Barcelona) The Barcelona Institute of Science and Technology Barcelona Spain; ^7^ Departament de Bioquímica i Biomedicina Molecular Facultat de Biologia Universitat de Barcelona Barcelona Spain

Dear Editor,

Metabolic dysfunction alters the properties of human adipose‐derived stem cells (hASCs), which are central to adipose tissue (AT) homeostasis and might also influence tumour microenvironments.^1^ Therefore, new knowledge of the secretory capacity of hASCs would be important not only in terms of AT physiology, but might also provide insights into tumorigenesis. This is particularly relevant for diabetes, as several studies have demonstrated that patients with type 2 diabetes (T2D) are at increased risk of developing several different types of cancer.^2^, ^3^ In the present study, we assessed the protein secretome of hASCs in a background of T2D (independent of obesity) using an untargeted proteomic analysis, and its potential relevance for their pro‐tumoral activity (Supporting Information). Our study reveals the importance of hASCs not only in metabolic disturbances, but also in tumorigenesis.

The secretome profiling of hASCs isolated from the subcutaneous AT of subjects with and without T2D was performed. Characteristics of the donors are summarized in Table S1. We observed that the hASC secretomes from the two groups displayed a different profile with good protein coverage (Figure S1A,B). Notably, global protein secretion was higher in T2D‐derived hASCs than in control hASCs (*p* < 0.039) (Figure S1A). Sample clustering and principal component analysis showed that one of the control samples grouped with the T2D samples and it was removed from the analysis (Figure S1C,D). The exploratory study was based on proteins detected in at least five of the six independent samples of the two groups, which was established to focus on traceable proteins. From this analysis, we observed a total of 231 unique proteins, of which 52 were identified to be differentially secreted (Figure 1A–C); 42 were secreted more by T2D‐derived hASCs (81% of all differentially secreted proteins) and 10 were secreted less by T2D‐derived hASCs (19%).

**FIGURE 1:**
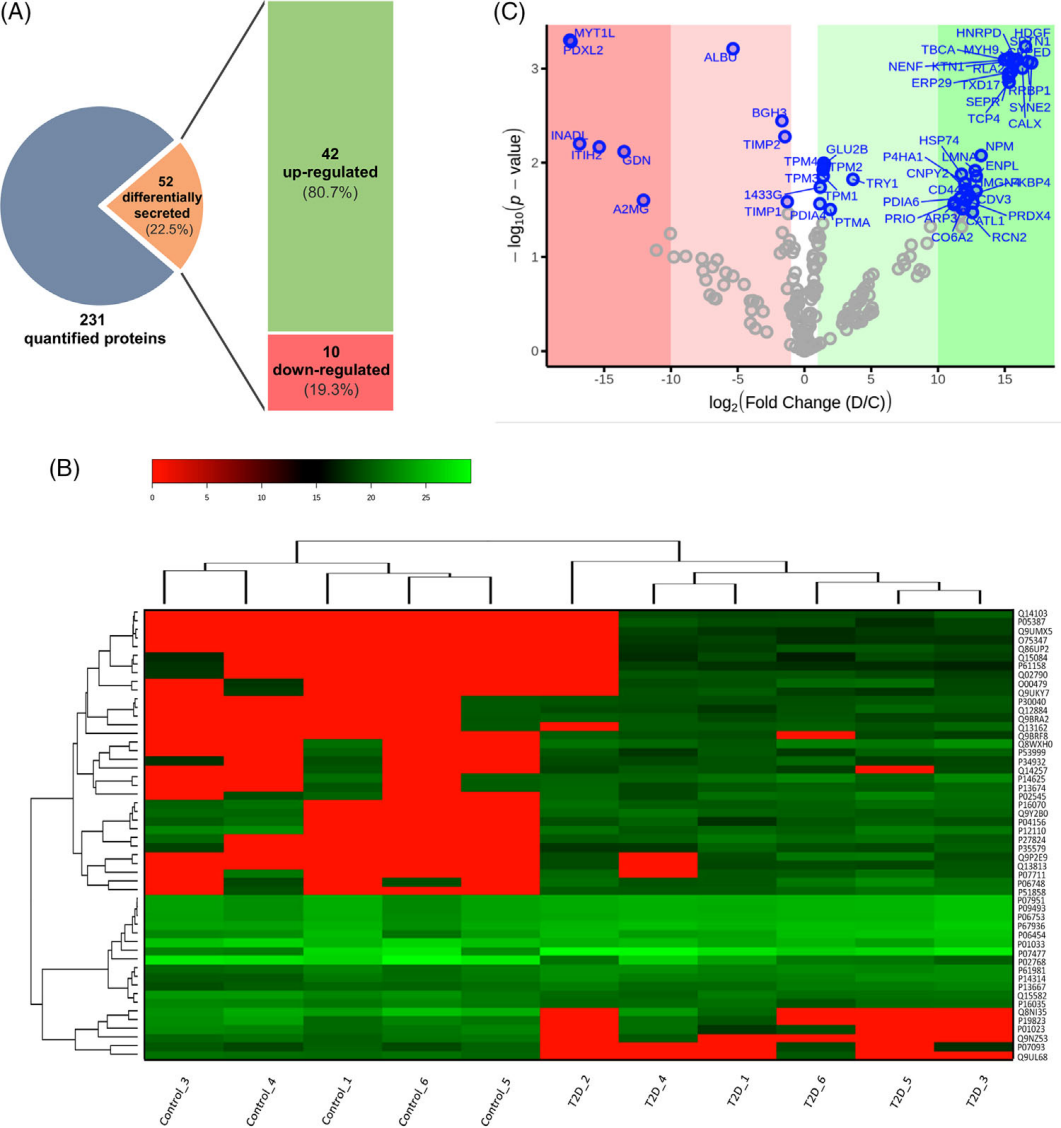
Differential expression analysis of secreted proteins in T2D‐hASCs. (A) Graphical representation of identified proteins. (B) Heat map of the differentially expressed proteins. Samples are in the columns and proteins in the rows. Uniprot IDs for each protein are shown at the right. Dendrograms show sample and protein clustering. (C) Volcano plot of all differentially expressed proteins; *x*‐axis shows the fold change between the two conditions (T2D/control) in log scale (base 2) and *y*‐axis the negative logarithm (base 10) of the *p*‐value. Proteins with calculated false discovery rate (FDR) < 0.05 are shown in blue. The intensity of the expression change is shown in green (higher in T2D samples) and red (lower in T2D samples)

The majority of secretory proteins are transported by the “conventional” secretion pathway to the plasma membrane, which involves the endoplasmic reticulum (ER) and the Golgi complex and is dependent on a signalling peptide; however, a significant number of proteins reach the plasma membrane/extracellular space via unconventional protein secretion (UPS) pathways independent of a signalling peptide.^4^ The 231 identified secreted proteins were examined for the presence of an N‐terminal signalling peptide by in silico functional analysis,^5^ which revealed that only 46.7% of the proteins were predicted to be released through the conventional trafficking pathway. Of the 10 proteins secreted less by T2D‐derived hASCs, 8 (80%) were predicted to be secreted by the conventional pathway. By contrast, only 19 of the 42 (45.2%) proteins secreted more by T2D‐derived hASCs were predicted to be transported by the conventional pathway. This finding suggests that UPS pathways are triggered by pathological conditions.

Functional analysis using STRING showed that differentially secreted proteins were associated with unique biological processes. Thus, network analysis of the proteins secreted more by T2D‐derived hASCs revealed a significant protein–protein interaction (PPI) enrichment of the network (Figure 2A). We focused on the more enriched pathways, based on the obtained *p*‐values in the curated databases, which included pathways associated with ER stress, extracellular matrix remodelling, cell adhesion and immune system, which are all directly related to cancer development and progression (Figure 2B).^6^ Network analysis of the proteins secreted less by T2D‐derived hASCs indicated that these proteins were associated with wound healing and with a variety of protease inhibitor events (Figure 2C,D).

**FIGURE 2:**
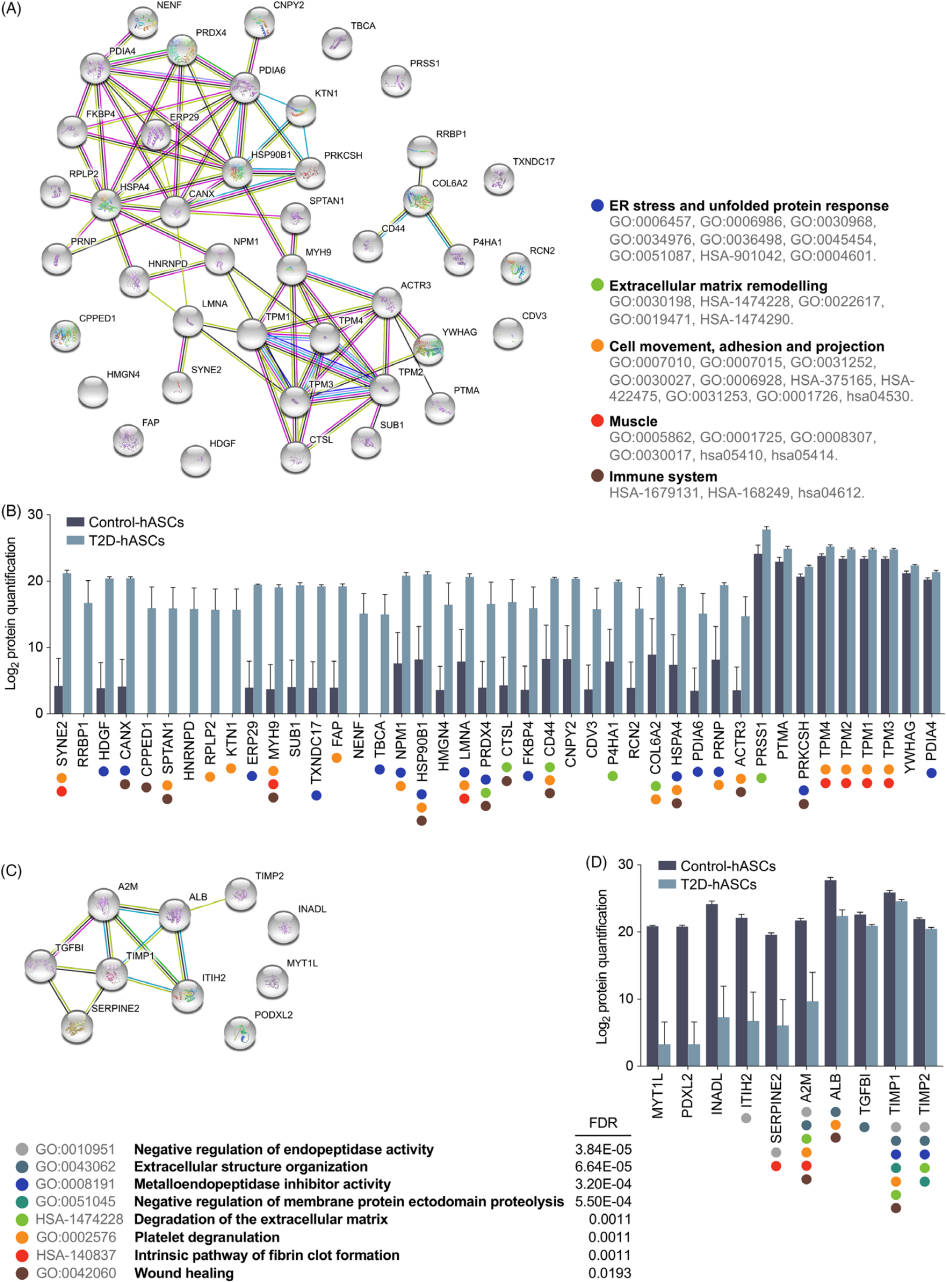
Network analysis and functional enrichment of the differentially secreted proteins in T2D‐hASCs. (A) Interaction network of the 42 proteins secreted more in the secretome of T2D‐hASCs from STRING analysis, showing a high degree of interaction between proteins (protein–protein interaction enrichment *p*‐value < 1.0 × 10^−16^). (B) Quantification values of the up‐secreted proteins ordered by significance and their enrichment into significant functional annotations retrieved from online databases. (C) Protein–protein interaction network of differentially expressed proteins under‐secreted in T2D‐hASCs from STRING analysis, showing a high degree of interaction between proteins (protein–protein interaction enrichment *p*‐value 3.47 × 10^−7^). (D) Quantification values of the under‐secreted proteins ordered by significance and their enrichment into significant functional annotations retrieved from online databases. Data was obtained from Ontology (GO#), Reactome (HSA‐#) and KEGG (hsa#) online databases (on 29 March 2019 through the STRING database version 11.0, https://string‐db.org). FDR < 0.05 was considered significant for the functional enrichment annotations

Because AT (and by extension hASCs) play an important role in T2D and associated co‐morbidities,^7^ the proteins secreted more by T2D‐derived hASCs can be considered as potential candidates in the pathophysiology of these diseases. To validate these putative biomarkers, we focused on the top‐15 over‐secreted proteins by fold change (Figure 3A). We used COMPARTMENTS, a unification and visualization tool of protein subcellular localization to identify proteins with multiple locations on the cellular organization (Figure 3B). This tool also confirmed the secretable peptides identified previously by in silico analysis.

**FIGURE 3:**
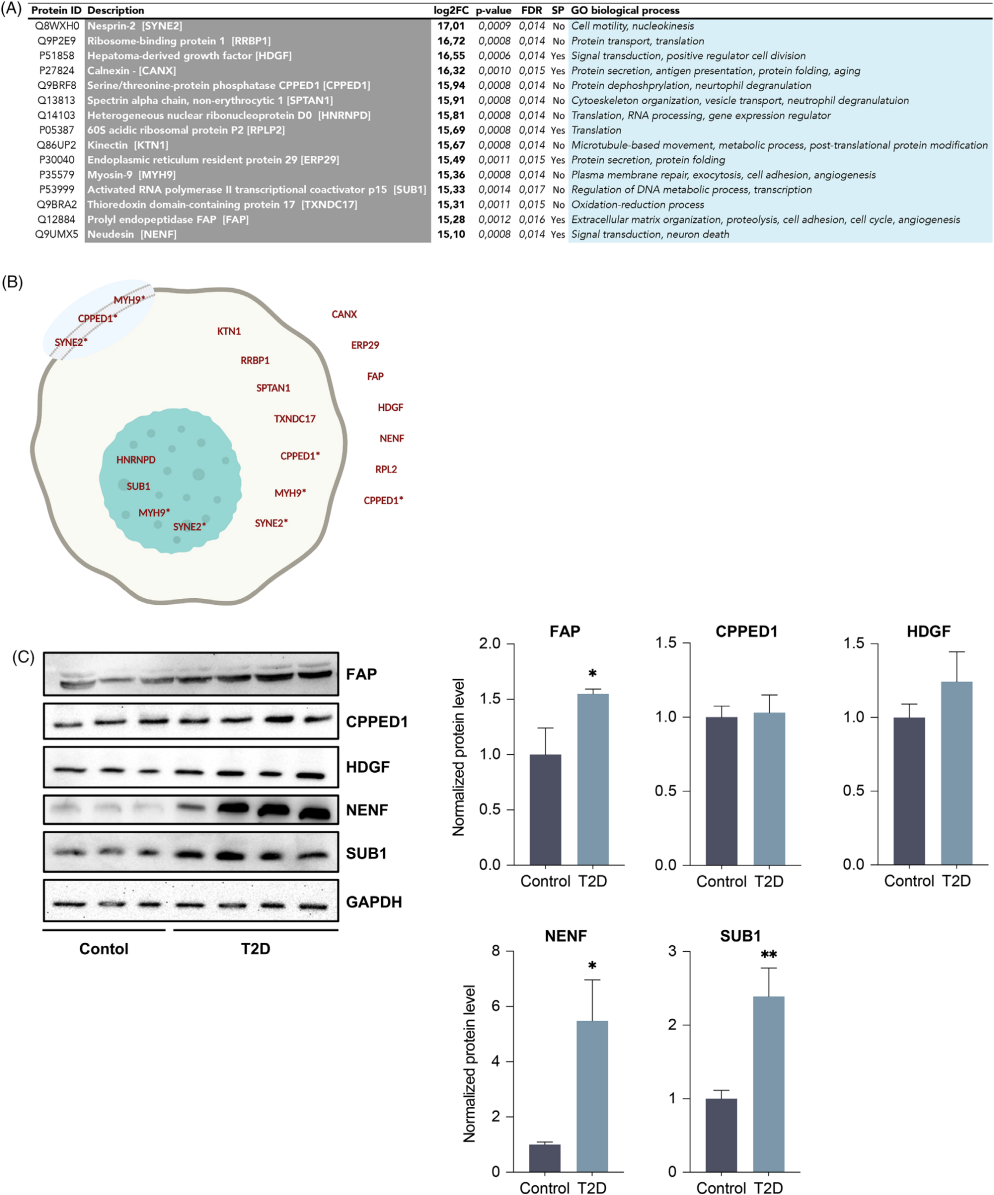
Study of the top‐15 over‐secreted proteins by T2D‐hASCs and validation of selected targets. (A) Table with the proteins arranged by log2 fold change (FC). The table includes the presence or absence of a signal peptide (SP) and biological processes. (B) Subcellular localization of the proteins (nuclear, cytosolic, membrane and extracellular compartments are depicted). Proteins marked with an asterisk (*) are proteins with multiple localizations. (C) Western blotting of CPEED, FAP, HDGF, NENF and SUB1 in control‐hASCs and T2D‐hASCs. GAPDH was used as a loading control. Representative images (proteins arranged by size) and densitometry analysis (arbitrary units) are shown (*n* = 8 per group). All values are expressed as mean ± SEM. **p < 0.01; *p < 0.05 versus control (paired Student's *t*‐test)

Remarkably, our proteomic approach allowed us to identify specific secreted proteins that could act as common determinants of diabetes and cancer. We analysed the steady‐state protein levels of CPPED1 (immune system pathway); fibroblast activation protein, FAP (movement, adhesion and projection); and hepatoma‐derived growth factor, HDGF (ER stress and unfolded protein response) by western blotting. We also assessed the levels of activated RNA polymerase II transcriptional coactivator p15 (SUB1) and neudesin (NENF), which were not enriched in any of the main identified pathways, but are known to have an important role in tumorigenesis and metabolism.^8^, ^9^ Protein expression analysis validated three of the selected targets in whole hASCs extracts: FAP, NENF and SUB1 (Figure 3C). NENF has been previously described to be elevated in patients with T2D, and seems to be related to obesity and insulin resistance,^10^ so was not considered for further analysis.

To test whether the diabetic secretome could modulate tumoral phenotypes we cultured human liver carcinoma (HepG2) cells for 24 h with CM from either T2D‐derived hASCs or control‐hASCs (Figure 4A), and we surveyed the expression of a panel of genes involved in inflammation, EMT, angiogenesis and invasiveness. We found that the expression of genes related to inflammation (*TNFA*), invasiveness (*MMP2* and *MMP9*) and tumour growth and metastasis (*VIM*), was significantly higher in HepG2 cells cultured in the CM of T2D‐hASCs than of control‐hASCs (Figure 4A). We repeated the gene expression analysis in HepG2 cells using neutralizing antibodies to FAP or SUB1. Blocking SUB1 (Figure 4B) but not FAP (Figure S2 reverted the tumoral phenotype induced by T2D‐hASC CM, as revealed by the significant downregulation of genes involved in inflammation (*IL1B*, *TNFA*), angiogenesis (*VEGFA*) and invasiveness (*MMP9*). Notably, neutralization of SUB1 also completely blocked the invasive properties of HepG2 cells in response to the T2D‐hASC secretome (Figure 4C). Overall, these data establish the involvement of secreted factors derived from hASCs in a diabetes microenvironment in enhancing the malignancy of cancer cells, and how, specifically, SUB1 might promote cell switching to a tumoral phenotype.

**FIGURE 4:**
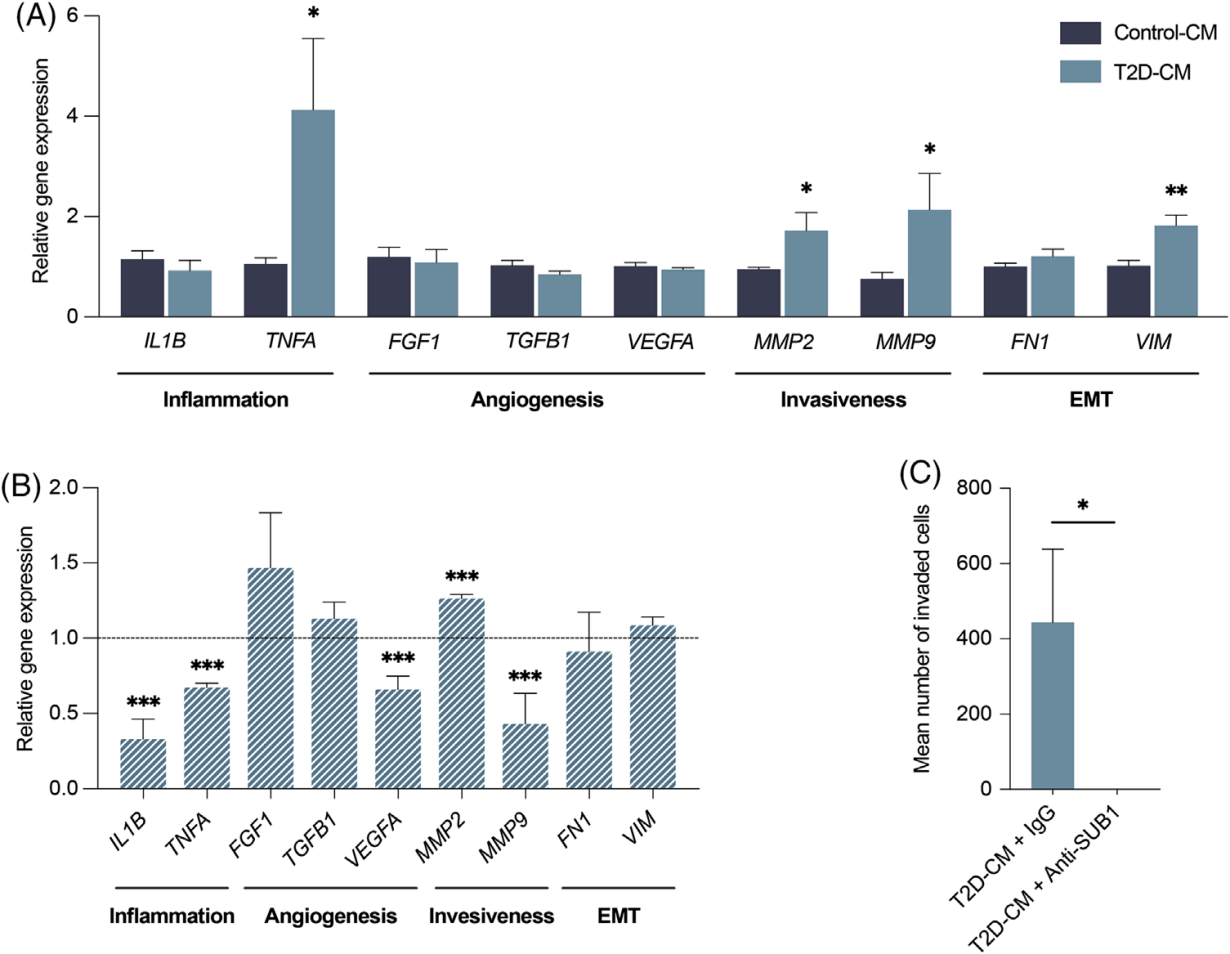
SUB1 neutralization blocks cancer‐related signaling and invasion in HepG2 cells. (A) Gene expression analysis of HepG2 cells cultured in CM of control‐hASCs or T2D‐hASCs. (B) Gene expression analysis of HepG2 cells cultured with CM of T2D‐hASCs in the presence of a SUB1 neutralizing antibody or an IgG control (20 mg IgG/ml). Control values (IgG) were set to 1 (dotted line). (C) Transwell invasion assay of HepG2 cells (*n* = 6 per group). Data are presented as mean ± SEM. *p < 0.05; **p < 0.01; ***p < 0.001 versus respective control (paired Student's *t*‐test)

In conclusion, our study indicates that research into the systemic effects of the adipose‐derived secretome may yield important insights into specific channels of systemic communication between the AT and tumours. We postulate that in a diabetic setting, hASCs acquire a pro‐tumoral secretome, including an over‐representation of SUB1, a secreted factor that can be internalized by surrounding cells to promote tumour progression. Further research will be needed to gain a better understanding of the molecular mechanisms underlying the interaction between components of the tumour microenvironment, and how such complex communication networks might be promoted in pathological environments such as those related to metabolic disorders. Finally, future preclinical studies should confirm that targeting SUB1 in vivo can modulate and prevent cancer progression.

## CONFLICT OF INTEREST

The authors declare no conflict of interest.

## Supporting information

Supporting information.

Supporting information.
