# Supplementary material for: Diabetes alters the protein secretome of human adipose‐derived stem cells and promotes tumorigenesis in hepatic cancer cells
Source: Clin Transl Med. 2022 Jun 2;12(6):e823. doi: 10.1002/ctm2.823 (PMC9161878; doi:10.1002/ctm2.823)
Supplement: Supplementary file 1 — Supporting information. [file CTM2-12-e823-s001.pdf]

Supplementary Information

MATERIALS AND METHODS

Study selection and sample processing

hASCs were obtained from the SAT of age- and sex-matched donors undergoing non-acute surgical interventions, such as hernia or cholecystectomy, in a scheduled routine surgery (n=6 per group) using standard protocols.[1–4] Briefly, SAT was washed extensively with phosphate buffered saline (PBS) and treated with 0.1% collagenase in PBS+1% bovine serum albumin (BSA) for 1 hour at 37ºC with agitation. Digested samples were centrifuged to separate adipocytes from stromal cells. The cell pellet containing the stromal fraction was resuspended in stromal culture medium. The medium was replaced 24 hours after seeding to remove non-adherent cells. When cultures reached approximately 80% confluency at passage 0 (P0), the cells were harvested by trypsin and seeded for the following passage or centrifuged to resuspend the pellet at a concentration of 10^6 hASCs/ml in cryomedium (10% DMSO, 10% DMEM/F-12, 80% bovine calf serum). The cells were dispensed in 1-ml aliquots in cryovials, maintained overnight in a -80ºC freezer and then stored in liquid nitrogen until they were required for the individual experiments. All participants gave their informed consent and the study was reviewed and approved by the ethics and research committee of University Hospital Joan XXIII, Tarragona, Spain. Clinical and laboratory data of the participants are shown in **Table S1**.

Adipose derived-stem cell culture and conditioned media collection

Individual vials of cells were removed from liquid nitrogen, placed in a 37ºC water bath and agitated rapidly but gently until the ice crystals were thawed. Cells were transferred to a 15-ml conical tube containing 5 ml of stromal medium consisting of DMEM/F12, 10% FBS, and 1% antibiotics (penicillin and streptomycin). The cell suspension was centrifuged at 1,200 rpm at room temperature, the supernatant aspirated to remove residual DMSO, and the cell pellet resuspended in stromal medium in

preparation for plating. Cell suspensions (P1) were seeded into T-75 cm^2 flasks. Cultures were replenished with fresh stromal medium every 2–3 days as described,[4] and cells were used for experiments from P3. Flow cytometry analysis of cell marker expression revealed that hASCs showed minimal functional and quantitative criteria as established by the International Society of Cell Therapy and the International Federation for Adipose Therapeutics and Science (Positive for CD73, CD90, CD105, and negative for CD14, CD31, CD34, CD45).

The used CM was collected from hASC cultures at P6 as follows: cells were plated at a density of 12,000 cells/cm^2 in 35-mm culture plates and allowed to grow for 24 hours. After this, the plates were first washed with PBS without calcium or magnesium, and then stromal medium without FBS was added to the cells. After 9 h, the CM was collected and frozen at -80ºC until required.

Conditioned medium processing for LC-MS/MS analysis

Samples were reduced with dithiothreitol (30 nmol, 37ºC, 60 min) and alkylated in the dark with iodoacetamide (60 nmol, 25ºC, 30 min). The resulting protein extract was first diluted to 2M urea with 200 mM NH_4HCO_3 for digestion with the endoproteinase LysC (1:10 w:w, 37ºC, o/n, cat #129-02541, Wako), and then diluted 2-fold with 200 mM NH_4HCO_3 for trypsin digestion (1:10 w:w, 37ºC, 8 h, cat #V5113, Promega). After digestion, the peptide mix was acidified with formic acid and desalted on a MicroSpin C18 column (The Nest Group, Inc.) prior to LC-MS/MS analysis.

LC-MS/MS analysis

Samples were analyzed in an LTQ-Orbitrap Velos Pro mass spectrometer (Thermo Fisher Scientific) coupled to a nano-LC (Proxeon) equipped with a reversed-phase chromatography 25-cm column with an inner diameter of 75 µm, packed with 1.9-µm C18 particles (Nikkyo Technos Co. Ltd.). Chromatographic gradients run were: 97% buffer A, 3% buffer B to 65% buffer A, 35% buffer B in 120 min at a flow rate of 250 nl/min (buffer A: 0.1% formic acid in water and buffer B: 0.1% formic acid in

acetonitrile). The instrument was operated in DDA mode and full MS scans with 1 micro scans at resolution of 60,000 were used over a mass range of m/z 350-2,000 with detection in the Orbitrap. Following each survey scan the top twenty most intense ions with multiple charged ions above a threshold ion count of 5000 were selected for fragmentation at normalized collision energy of 35%. Fragment ion spectra produced via collision-induced dissociation (CID) were acquired in the linear ion trap. Digested bovine serum albumin (cat. #P8108S, New England Biolabs) was analyzed between each sample to avoid sample carryover and to assure stability of the instrument, and Qcloud[5] was used to control instrument longitudinal performance during the project. All data were acquired with Xcalibur software v2.2. The mass spectrometry proteomics data have been deposited to the ProteomeXchange Consortium via the PRIDE partner repository[6] with the dataset identifier PXD029829.

Secretome data analysis

Acquired spectra were analyzed using the Proteome Discoverer software suite (v1.4, Thermo Fisher Scientific) and Mascot search engine v2.5 (Matrix Science).[7] Data were searched against the Swiss-Prot human database (release date April 2018) plus a list of common contaminants (refined from MaxQuant contaminants.fasta) and the corresponding decoy entries. For peptide identification, a precursor ion mass tolerance of 7 ppm at the MS1 level was used, trypsin was chosen as enzyme, and up to three missed cleavages was allowed. The fragment ion mass tolerance was set to 0.5 Da for MS2 spectra. Oxidation of methionine and N-terminal protein acetylation were defined as variable modifications, whereas carbamidomethylation on cysteines was set as a fixed modification. FDR for peptide identification was set to a maximum of 5%.

Protein abundance was estimated using the average area under the chromatographic peak for the three most intense peptides. Differential enrichment was done using the DEP package, which provides an integrated analysis workflow for robust and reproducible analysis of MS proteomics data.[8] Briefly, identified proteins were filtered to keep those detected in all or all but one replicate of at least one condition, resulting in 231 proteins retained. The filtered data were then background corrected and

normalized by variance stabilizing transformation.[9] Protein-wise linear models combined with empirical Bayes statistics were used for the differential enrichment analysis using the *limma* package.[10]

In silico protein functional analysis

Computational prediction of secreted proteins was performed using SignalP (http://www.cbs.dtu.dk/services/SignalP/) and by UniProt including proteins with the keywords "Signal," "Secreted," or "Extracellular space." If a protein belonged to at least one of these four criteria, it was considered "predicted to be secreted" otherwise "not predicted to be secreted". Known interactions between ligands and receptors were retrieved from MetaCore. Plasma membrane receptors were defined as those having either the UniProt keyword "cell membrane" or Panther protein class "receptor" or GO.CC "plasma membrane". Self-interactions were discarded.[11] COMPARTMENTS, a unification and visualization tool of protein subcellular localization based on evidence (https://compartments.jensenlab.org/), was used to identify proteins with multiple locations and to depict differentially-expressed proteins on the cellular organization.[12] For functional studies, *STRING v11: protein–protein association networks* (https://string-db.org/) was used to evaluate the implicated molecular and functional pathways for the candidate genes.

Western blotting

Equal amounts of protein were subjected to SDS-PAGE, transferred to immobilon membranes and blocked. Immunoreactive bands were visualized using the SuperSignal West Femto chemiluminescent substrate (Pierce) and images were captured using the VersaDoc Imaging System and Quantity One software (Bio-Rad). The following antibodies were used: anti-CPPED1 (sc-514222), anti-HDGF (sc-271344), and anti-SUB1 (sc-166280) from Santa Cruz Biotechnology; anti-FAP (ab28244) from Abcam; anti-GAPDH (G8795) from Sigma-Aldrich; and anti-NENF (#24736) from SAB Signalway Antibody Inc. For all experiments, antibody dilutions were 1/1000 and incubation was performed at 4ºC overnight.

HepG2 cell culture

The HepG2 liver cancer cell line was obtained from the ATCC. Cells were propagated in DMEM/F12 supplemented with 1% L-glutamine (Sigma-Aldrich), 10% FBS, 1% antibiotics/antimycotics solution and 2% HEPES (HyClone) at a density of 40,000 cells/cm^2. Cells were cultured in a humidified incubator at 37ºC with 5% CO_2. Experiments using CM were performed using 24-h cultures.

Transwell invasion assays

The invasion capacity of cancer cells in response to 24-h application of CM from hASCs was determined using 24-well Transwells (#3422, Costar), as described.[4] In total, 2×10^5 HepG2 cells were suspended in 200 µl CM and added to the upper chamber, and 500 µl culture medium was placed in the lower chamber. The membrane was first coated with Matrigel® (0.7–0.9 mg/ml; Sigma- Aldrich) in PBS for 2 h at 37ºC. After 24 h incubation, the cells in the upper compartment were removed using cotton swabs, after which the cells on the lower surface of the membrane were fixed in 4% glutaraldehyde, stained with 2% toluidine blue and counted.

SUB1 blocking experiments

SUB1 neutralization was performed by adding 20 µg/ml of an anti-SUB1 antibody (sc-166280, Santa Cruz Biotechnology) to the ASCs CM for 1 h at room temperature before adding the medium to HepG2 cells. A negative epitope control (mouse IgG Isotype Control, Invitrogen) was included in each experiment.

Gene expression analysis

Total RNA was extracted from cells using TRI Reagent (Molecular Research Center). Quantification was performed at 260 nm and purity was assessed by the OD260/OD280 ratio. For gene expression analysis, 1 µg RNA was reverse-transcribed with random primers using a Reverse Transcription System (Applied Biosystems). Quantitative RT-PCR (qRT-PCR) was conducted on a ProFlex PCR System using TaqMan

Gene Expression Assays (Applied Biosystems) (**Table S2**). Results were calculated using the comparative Ct method (2-ΔΔCt) normalized to the expression of the housekeeping gene 18S (Hs 03928985_g1) and expressed relative to the control condition set to 1. Two technical duplicates were performed for each biological replicate.

Statistical analysis

In vitro experiments were performed 5–6 times and pooled for statistical analysis. Data are presented as mean ± S.E.M. and represent the number of biologically-independent samples. Differences between groups were determined using unpaired Student's t-test to compare two groups (two-tailed, 95% confidence interval). The analyses were performed using GraphPad Prism 8.0.2 software (GraphPad Software Inc.). A p-value < 0.05 was considered statistically significant. For clinical and anthropometrical variables, normally distributed data were expressed as mean ± S.D.

1. Yu G, Wu X, Dietrich MA, et al. Yield and characterization of subcutaneous human adipose-derived stem cells by flow cytometric and adipogenic mRNA analyzes. *Cytotherapy*. 2010;12(4):538-546. doi:10.3109/14653241003649528
2. Gimble JM, Guilak F. Differentiation potential of adipose derived adult stem (ADAS) cells. *Curr Top Dev Biol*. 2003;58:137-160. doi:10.1016/s0070-2153(03)58005-x
3. Shah FS, Li J, Zanata F, et al. The Relative Functionality of Freshly Isolated and Cryopreserved Human Adipose-Derived Stromal/Stem Cells. *Cells Tissues Organs*. 201(6):436-444. doi:10.1159/000446494
4. Serena C, Keiran N, Ceperuelo-Mallafre V, et al. Obesity and Type 2 Diabetes Alters the Immune Properties of Human Adipose Derived Stem Cells. *Stem Cells*. 2016;34(10):2559-2573. doi:10.1002/stem.2429
5. Chiva C, Olivella R, Borràs E, et al. QCloud: A cloud-based quality control system for mass spectrometry-based proteomics laboratories. *PLoS One*. 2018;13(1):e0189209.

doi:10.1371/journal.pone.0189209

6. Vizcaíno JA, Csordas A, Del-Toro N, et al. 2016 update of the PRIDE database and its related tools. *Nucleic Acids Res*. 2016;44(D1):D447-56. doi:10.1093/nar/gkv1145

7. Perkins DN, Pappin DJ, Creasy DM, Cottrell JS. Probability-based protein identification by searching sequence databases using mass spectrometry data. *Electrophoresis*. 1999;20(18):3551-3567. doi:10.1002/(SICI)1522-2683(19991201)20:18<3551::AID-ELPS3551>3.0.CO;2-2

8. Zhang X, Smits AH, Van Tilburg GBA, Ovaa H, Huber W, Vermeulen M. Proteome-wide identification of ubiquitin interactions using UbIA-MS. *Nat Protoc*. 2018;13(3):530-550. doi:10.1038/NPROT.2017.147

9. Välikangas T, Suomi T, Elo LL. A systematic evaluation of normalization methods in quantitative label-free proteomics. *Brief Bioinform*. 2018;19(1):1-11. doi:10.1093/BIB/BBW095

10. Ritchie ME, Phipson B, Wu D, et al. limma powers differential expression analyses for RNA-sequencing and microarray studies. *Nucleic Acids Res*. 2015;43(7):e47. doi:10.1093/NAR/GKV007

11. Okawa S, Gagrica S, Blin C, Ender C, Pollard SM, Krijgsveld J. Proteome and Secretome Characterization of Glioblastoma-Derived Neural Stem Cells. *Stem Cells*. 2017;35(4):967-980. doi:10.1002/stem.2542

12. Binder JX, Pletscher-Frankild S, Tsafou K, et al. COMPARTMENTS: unification and visualization of protein subcellular localization evidence. *Database (Oxford)*. 2014;2014:bau012. doi:10.1093/database/bau012
