# Supplementary material for: Diabetes alters the protein secretome of human adipose‐derived stem cells and promotes tumorigenesis in hepatic cancer cells
Source: Clin Transl Med. 2022 Jun 2;12(6):e823. doi: 10.1002/ctm2.823 (PMC9161878; doi:10.1002/ctm2.823)
Supplement: Supplementary file 2 — Supporting information. [file CTM2-12-e823-s002.pdf]

	Controls	T2D
**n**	6	6
**Sex (M/F)**	(4/2)	(2/4)
**Age (yrs)**	58.9 ± 5.9	62.6 ± 9
**BMI (kg/m2)**	32.2 ± 3.5	31.2 ± 5
**HOMA-IR**	2.9 ± 1.5	2.4 ± 2.5
**Cholesterol (mg/dl)**	216 ± 29.2	202 ± 30
**HDL (mg/dl)**	55 ± 6.7	46.4 ± 12.2
**LDL (mg/dl)**	138.3 ± 14.9	122.5 ± 37.5
**TG (mg/dl)**	113.8 ± 14.9	192.6 ± 87.1

Table S1. Clinical and laboratory data for the patients included in study.

Data are presented as mean ± SD. T2D, type 2 diabetes; BMI, body mass index; HOMA-IR, insulin resistance homeostatic model assessment. Differences were analysed by the unpaired *t*-test. *$p<0.05$ *vs* Control.

Table S2. Human gene expression Taqman probes. Results were calculated using the comparative Ct method and expressed relative to the expression of the housekeeping gene 18S.

**Detector**	**Taqman probes**	**Gene probes**
Immune regulators		
IL1b	Hs01555410_m1	Interleukin 1 Beta
TNF-α	Hs00174128_m1	Tumor necrosis factor alpha
Invasiveness		
MMP2	Hs01548727_m1	Matrix Metalloproteinase-2
MMP9	Hs00234579_m1	Matrix Metalloproteinase-9
VIM	Hs00958111_m1	Vimentin
FN1	Hs01549976_m1	Fibronectin
VEGFa	Hs00900054_m1	Vascular Endothelial Growth Factor A
TGF-β1	Hs00998133_m1	Transforming Growth Factor Beta 1
FGF1	Hs01092738_m1	Fibroblast growth factor 1
Housekeeping		
18S rRNA	Hs03928985_g1	18S ribosomal RNA

Figure S1

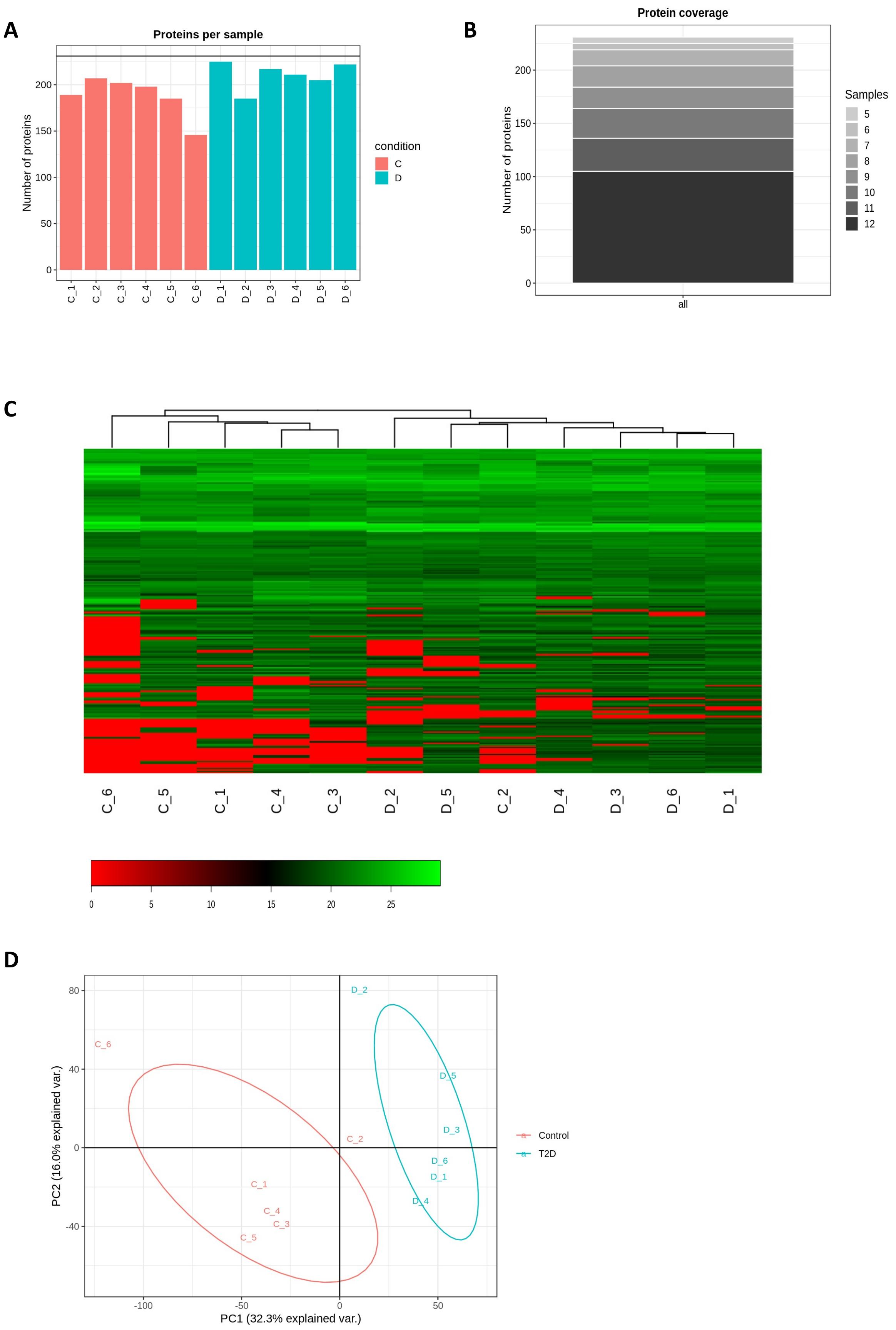

Figure S1. **Inspection data.** Exploratory analysis of proteins detected in at least 5 samples of a group (control or T2D). **A)** Number of proteins identified per sample, the black horizontal line indicates total number of identified proteins. **B)** Protein coverage, accumulated number of identified proteins per sample. **C)** Heat map of all identified proteins. Samples are in the columns and the identified proteins in the rows, dendrogram on top shows sample clustering. **D)** Principal Components Analysis (PCA) of all identified proteins.

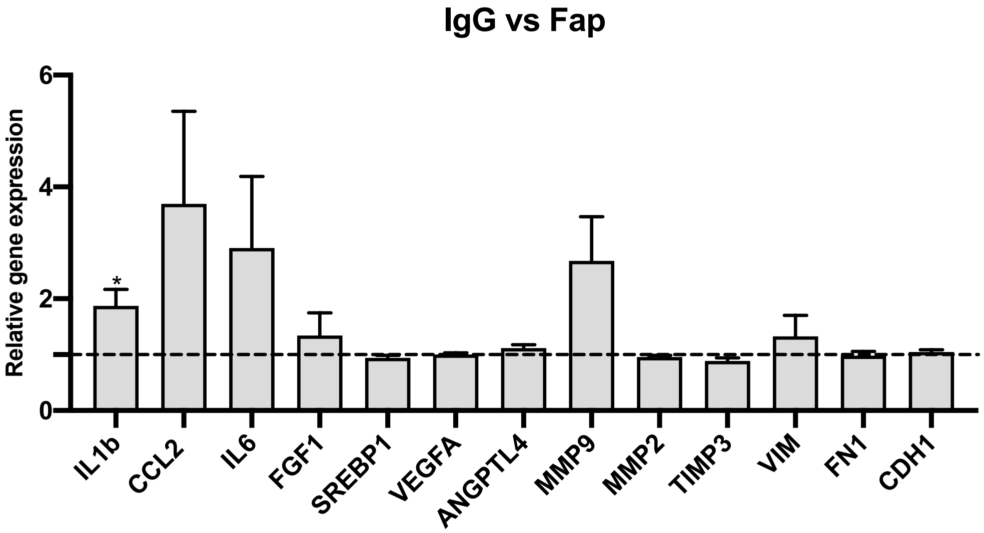

Figure S2. **FAP downregulation has no positive effects on HepG2 cancer cells**. Gene expression analysis of HepG2 cells cultured with CM T2D in the presence of a FAP neutralizing antibody or an IgG control (20 mg IgG/mL). Control values (IgG) were set to 1 (dotted line). n=6. Data are presented as mean ± SEM. *p < 0.05 versus respective control (paired Student's t test).
